# Catastrophic antiphospholipid syndrome complicated with multiple infections: a case report

**DOI:** 10.3389/fimmu.2026.1764711

**Published:** 2026-04-30

**Authors:** Lingli Zhou, Shuangshuang Liang, Huadong Zhu, Hua Chen, Jingjing Chai, Xiaojun Ma, Jing Yang

**Affiliations:** 1 Department of Emergency, State Key Laboratory of Complex Severe and Rare Diseases, Critical and Emergency Pharmaceuticals & Medical Devices Innovation Lab, Peking Union Medical College Hospital, Peking Union Medical College & Chinese Academy of Medical Sciences, Beijing, China; 2 Emergency Department, Zhengzhou Central Hospital, Zhengzhou, China; 3 Rheumatology and Immunology Department, Peking Union Medical College Hospital, Chinese Academy of Medical Science and Peking Union Medical College, Beijing, China; 4 Department of Infectious Diseases, Peking Union Medical College Hospital, Chinese Academy of Medical Sciences and Peking Union Medical College, Beijing, China

**Keywords:** autopsy, catastrophic antiphospholipid syndrome, infection, thrombosis, case report

## Abstract

**Background:**

Catastrophic antiphospholipid syndrome (CAPS) is a rare and life-threatening manifestation of antiphospholipid syndrome (APS), which is frequently later diagnosed with high mortality.

**Case report:**

A 54-year-old woman with a history of livedo reticularis presented with fever for four days, hematuria for two days, and impaired consciousness and petechiae for one day. Laboratory tests indicated thrombocytopenia, anemia, elevated indirect bilirubin and lactate dehydrogenase levels, as well as elevated free hemoglobin, isolated C3d positivity on direct antiglobulin test, and positive lupus anticoagulant and anti-cardiolipin IgG (aCL-IgG). A diagnosis of autoimmune hemolytic anemia and thrombocytopenia was established, and high-dose methylprednisolone combined with intravenous immunoglobulin therapy was initiated. On day 4, a tricuspid valve thrombus was detected and argatroban was initiated. Although the isolated LA positivity and low-titer aCL-IgG did not fulfill classic APS criteria, the definitive thrombosis led us to strongly suspect quasi-seronegative APS, prompting an increased methylprednisolone dose. On day 6, the platelet count dropped again, and a diagnosis of CAPS was suspected, prompting the administration of methylprednisolone pulse therapy along with plasma exchange. On day 6, the platelet count dropped again, and the patient received methylprednisolone pulse along with plasma exchange treatment. On day 10, leukopenia complicated mixed bacterial infections were developed. On day 11, subarachnoid hemorrhage occurred, leading to discontinuation of anticoagulant. Ultimately, the patient died with the worsened infections. Autopsy revealed extensive thrombosis and severe disseminated mixed infections.

**Conclusions:**

Early manifestations of CAPS may be atypical, so early identification is crucial for treatment. Following steroid pulse therapy, vigilance for fungal infections is essential, with empirical antifungal therapy initiated when necessary.

## Introduction

Approximately 1% of APS patients develop a severe clinical subtype characterized by multiple thromboses involving mainly small vessels, called catastrophic antiphospholipid syndrome (CAPS), which leads to multi-organ failure, with a mortality rate of 50% (1–5). Patients with clinical features highly suggestive of APS but persistently negative for standard antibodies are termed seronegative APS (SNAPS) (6–8). Like classic APS, SNAPS can also progress rapidly to life-threatening CAPS. Diagnosing CAPS is inherently challenging, especially in seronegative patients, as its pathophysiology, signs, and symptoms overlap with other hematologic conditions such as thrombotic microangiopathy (TMA) and immune thrombocytopenia (ITP), often leading to delayed treatment and poor outcomes. In this article, we presented a CAPS patient with clinical manifestations, laboratory tests, treatment response, and final clinical pathology discussion, to enhance the clinician’s understanding of catastrophic antiphospholipid syndrome.

## Case presentation

A 54-year-old female patient was admitted to the Emergency Intensive Care Unit with “fever for 4 days, hematuria for 2 days, and impaired consciousness and petechiae for 1 day”. She initially presented with fever (peak temperature 39.1 °C) and toothache, in the absence of respiratory symptoms. Two days later, she developed hematuria accompanied by lumbar back pain. She has an over-10-year history of livedo reticularis in bilateral calves with an unknown cause. The patient reports that the livedo typically worsens accompanied by a decrease in skin temperature when exposed to cold environments, but there was no documented acute exacerbation during the current admission. No specific treatment was received. There was no typical Raynaud’s phenomenon such as the sequence of white-purplish-red changes at the extremities. Besides, she had hypertension for 1-year, oral medication was taken and the blood pressure was controlled at around 120/90 mmHg. She has no history of obstetric morbidity, or documented history of thrombocytopenia, hemolytic anemia, or other hematological abnormalities prior to this admission.

Relevant preliminary laboratory evaluation from another hospital included a decreased hemoglobin (Hb) at 106 g/L (reference, 120–160 g/L), normal platelet (PLT) count at 115 × 10^9^/L (reference, 100-300 × 10^9^/L), 2+ proteinuria, 3+ hematuria, elevated procalcitonin (PCT) at 7.92 ng/mL (reference, <0.025 ng/mL), elevated C-reactive protein (CRP) at 58.88 mg/L (reference, <0.5 mg/L), elevated D-dimer at 66,822 ng/mL (reference, <243 ng/mL), elevated fibrin degradation products (FDP) >120 μg/mL (reference, 0–5 μg/mL), elevated lactate dehydrogenase (LD) at 3,685 U/L (reference, <250 U/L), elevated alanine aminotransferase (ALT) at 297 U/L (reference, <50 U/L), normal serum creatinine (Cr), and a positive direct antiglobulin test (DAT). Empiric therapy was started with meropenem and moxifloxacin. The patient was transferred to our emergency department with progressive clinical deterioration, subsequently developing rapidly declining consciousness, agitation, and severe respiratory distress requiring emergent endotracheal intubation.

On admission, vital signs showed a blood pressure of 114/78 mmHg, pulse rate of 109 beats/min, body temperature of 38.3°C, and respiratory rate of 21 breaths/min. Oxygen saturation was 100% at a respiratory oxygen concentration of 45%. Physical examination revealed mottling of the extremities and diminished bowel sounds.

The routine laboratory tests revealed moderate anemia (Hb 82 g/L) thrombocytopenia (PLT 32 × 10^9^/L), hemolytic profile (total bilirubin 28.7 μmol/L, direct bilirubin 12.4 μmol/L, LD 5,263 U/L), systemic inflammation (hyper-sensitive C-reactive protein 105.8 mg/L, PCT 11 ng/mL, D-dimer 29.74 mg/L, Fibrinogen 5.74 g/L) and multi-organ dysfunction (ALT 211 U/L, Cr 151 μmol/L, B-natriuretic peptide 3,989 ng/L, creatine kinase-MB 38.1μg/L, hyper-sensitive troponin I 20,637 ng/L, Myoglobin 1,809 μg/L). Other blood tests showed normal range for white blood cell count, neutrophils, albumin, prothrombin time and activated partial thromboplastin time. The laboratory test results are shown in **Table 1**. A lumbar puncture was performed, with a cerebrospinal fluid pressure of 265 mmH_2_O and unremarkable routine and biochemical tests of cerebrospinal fluid. The initial chest computed tomography (CT) scan and contrast-enhanced abdominopelvic CT scan showed no significant abnormal findings.

**Table 1:** Patient’s laboratory evaluation.

Variables	On admission	Reference range
White blood cell count(×10^9^/L)	5.76	3.5-9.5
Neutrophils(×10^9^/L)	3.92	2.0-7.5
Hemoglobin (g/L)	82	120-160
Platelet count(×10^9^/L)	32	100-300
Alanine aminotransferase (U/L)	211	9-50
Albumin (g/L)	35	35-52
Total bilirubin (μmol/L)	28.7	5.1-22.2
Direct bilirubin (μmol/L)	12.4	≤6.8
Lactate dehydrogenase (U/L)	5,263	0-250
Creatinine (μmol/L)	151	59-104
Urea nitrogen (mmol/L)	16.82	2.8-7.2
hyper-sensitive C-reactive protein (mg/L)	105.8	<3
Procalcitonin (ng/mL)	11	≤0.025
B-natriuretic peptide (ng/L)	3,989	0-100
Creatine Kinase-MB (μg/L)	38.1	≤5
hyper-sensitive troponin I (ng/L)	20,637	≤54
Myoglobin (μg/L)	1,809	≤110
Prothrombin time (s)	12.2	10.4-12.6
Activated partial thromboplastin time (s)	29	23.3-32.5
D-dimer (mg/L)	29.74	0-0.55
Fibrinogen (g/L)	5.74	1.8-3.5

**Figure 1:**
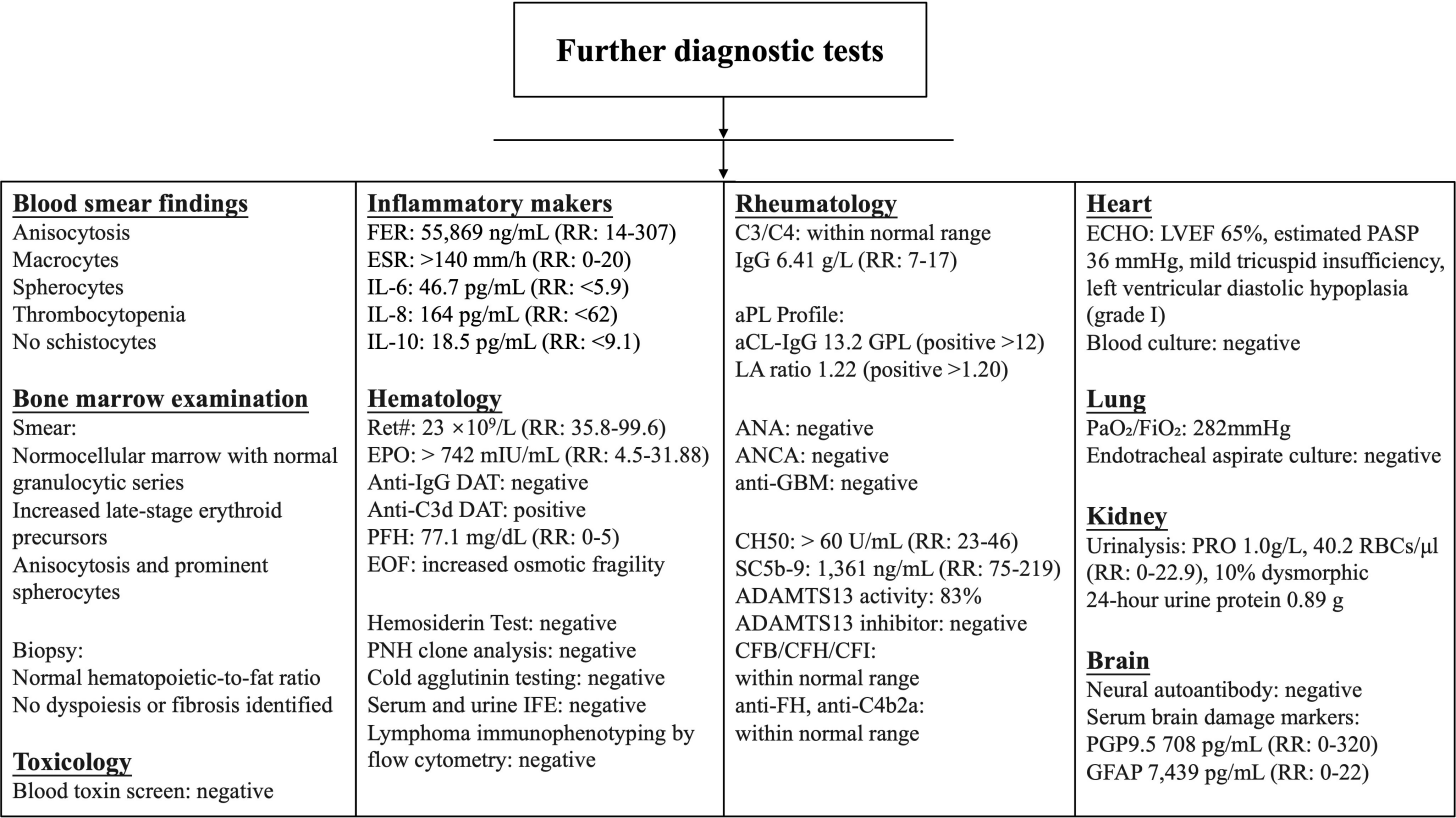
Extensive workup during hospitalization. aCL, Anti-Cardiolipin Antibody; ADAMTS13, A Disintegrin and Metalloproteinase with Thrombospondin Type 1 Motif, 13; ANA, Antinuclear Antibody; ANCA, Anti-Neutrophil Cytoplasmic Antibody; anti-C4b2a, Anti-C3 Convertase (C4b2a) Antibody; anti-FH, Anti-Factor H Autoantibody; anti-GBM, Anti-Glomerular Basement Membrane Antibody; aPL, Antiphospholipid Antibody; C3, Complement C3 level; C4, Complement C4 level; CFB, Complement Factor B; CFH, Complement Factor H; CFI, Complement Factor I; CH50, Complement Hemolysis 50; DAT, Direct Antiglobulin Test; ECHO, Echocardiography; EOF, Erythrocyte Osmotic Fragility; EPO, Erythropoietin; ESR, Erythrocyte Sedimentation Rate; FER, Ferritin; GFAP, Glial Fibrillary Acidic Protein; GPL, Gamma Phospholipid Lunits, IFE, Immunofixation Electrophoresis; IgG, ImmunoglobulinG; IL, Interleukin; LA, Lupus Anticoagulant; LVEF, Left Ventricular Ejection Fraction; PASP, Pulmonary Artery Systolic Pressure, PFH, Plasma Free Hemoglobin; PGP9.5, Protein Gene Product 9.5; PNH, Paroxysmal Nocturnal Hemoglobinuria; Ret#, Reticulocyte count; RR, Reference Range; SC5b-9, Soluble Complement 5b-9 Complex.

As the diagnosis remained unclear, an extensive workup was performed and shown in **Figure 1**. The peripheral blood smear demonstrated anisocytosis, macrocytes, and spherocytes with thrombocytopenia, in the absence of schistocytes. The bone marrow examination revealed no clinically significant abnormalities. The blood toxicological screen was negative. The diagnosis of systemic hyperinflammation was confirmed by hyperferritinemia (55,869 ng/mL), extremely elevated erythrocyte sedimentation rate (> 140 mm/h), and elevated cytokine profiling (IL-6 46.7 pg/mL, IL-8–164 pg/mL, IL-10 18.5 pg/mL). The hematologic workup revealed decreased eticulocyte count (23 ×10^9^/L), extremely elevated erythropoietin (> 742 mIU/mL), positive anti-C3d DAT, elevated plasma free hemoglobin (77.1 mg/dL), and increased osmotic fragility. Other specialized hematologic investigations returned negative results, including hemosiderin test, paroxysmal nocturnal hemoglobinuria (PNH) clone analysis, cold agglutinin testing, serum and urine immunofixation electrophoresis (IFE), and lymphoma immunophenotyping by flow cytometry. The rheumatologic workup revealed decreased IgG (6.41g/L), dual positivity of anti-cardiolipin antibody (aCL-IgG 13.2 GPL) and lupus anticoagulant (LA detected by dilute Russell’s viper venom time [dRVVT] with screen/confirm ratio of 1.22; laboratory cut-off for positivity: >1.20), extremely elevated Complement Hemolysis 50 (CH50 > 60 U/mL), significantly elevated Soluble Complement 5b-9 Complex (SC5b-9–1,361 ng/mL), and 83% of ADAMTS13 activity. Specialized rheumatologic investigations returned negative results, including antinuclear antibody (ANA), anti-neutrophil cytoplasmic antibody (ANCA), and anti-glomerular basement membrane antibody (anti-GBM). Other results were within normal range, such as C3, C4, ADAMTS13 inhibitor, complement factors (CFB, CFH and CHI), anti-factor H autoantibody (anti-FH) and anti-C3 convertase (C4b2a) antibody. Echocardiography revealed normal left ventricular ejection fraction (LVEF 65%), right ventricular dysfunction and elevated pulmonary artery systolic pressure (estimated 36 mmHg). Both echocardiography and blood cultures showed no evidence of infective endocarditis. The PaO_2_/FiO_2_ was 282mmHg and no significant bacterial or fungal pathogens were isolated from the endotracheal aspirate. Urinalysis revealed proteinuria (1.0 g/L) with microscopic hematuria (40.2 RBCs/μL) including 10% dysmorphic RBCs, and 24-hour urine protein was 0.89 g. Neural autoantibody testing was negative, while serum brain damage markers showed significant elevations: protein gene product 9.5 (PGP9.5–708 pg/mL) and glial fibrillary acidic protein (GFAP 7,439 pg/mL). In addition, CT pulmonary angiography (CTPA) and systemic vascular ultrasound (including deep veins and arteries of the upper and lower extremities, portal venous system, superior mesenteric vein, splenic vein, and renal veins) revealed no definite evidence of thrombosis.

**Figure 2:**
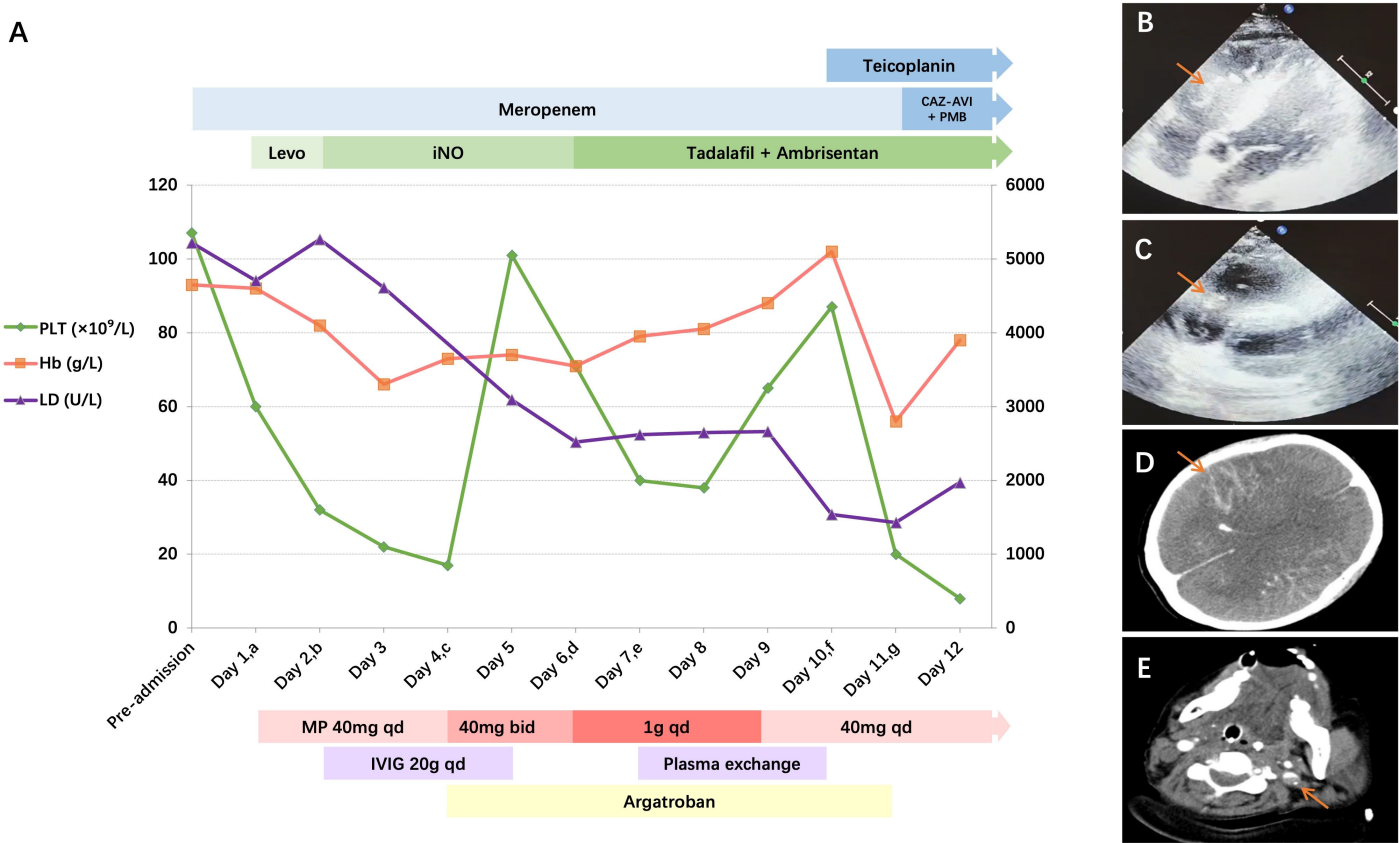
Diagram of disease course and treatment. **(A)** The trends of critical laboratory results, significant clinical events, and main therapeutic interventions. (a) On admission, initial treatment included MP 40mg qd, levosimendan, and meropenem. (b) Day 2, IVIG 20g qd was added and iNO was initiated for pulmonary hypertension. (c) Day 4, transthoracic echocardiography revealed right ventricular thrombus, MP was adjusted to 40mg bid and argatroban was initiated. (d) Day 6, MP 1g qd pulse therapy started for the suggested diagnosis of CAPS. Pulmonary hypertension targeted therapy was transited to oral tadalafil and ambrisentan. (e) Day 7, plasma exchange was initiated to intensify treatment. (f) Day 10, teicoplanin was added due to persistent fever and neutropenia. (g) Day 11, the regimen was adjusted to targeted therapy with ceftazidime-avibactam (for MDR *K. pneumoniae*), polymyxin B (for *A. baumannii*), and continued teicoplanin (for *Enterococcal* bacteremia). CTA demonstrated acute subarachnoid hemorrhage, left internal carotid artery thrombosis, tonsillar herniation. Anticoagulation was discontinued. **(B, C)** Transthoracic echocardiography revealed right ventricular thrombus (orange arrow). **(D)** CTA demonstrated acute subarachnoid hemorrhage (orange arrow). **(E)** CTA demonstrated left internal carotid artery thrombosis (orange arrow). Bid, twice daily; CAZ-AVI, ceftazidime-avibactam; Hb, hemoglobin; iNO, inhaled nitric oxide; IVIG, intravenous immunoglobulin; LD, lactate dehydrogenase; Levo, levosimendan; MP, methylprednisolone; PLT, platelet; PMB, polymyxin B; qd, once daily.

**Figure 3:**
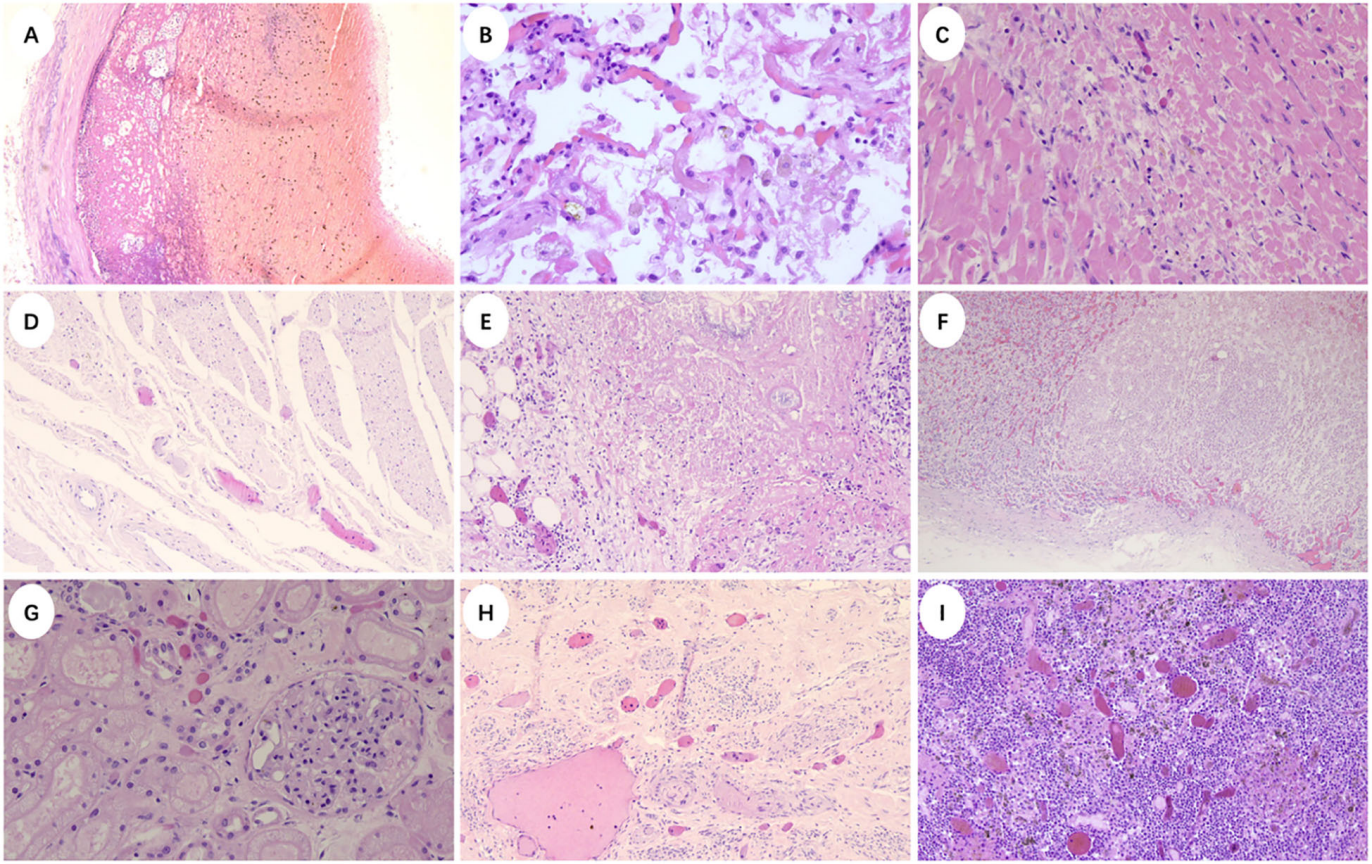
Clinicopathological findings of multi-organ thrombosis. Thrombosis of **(A)** Left middle cerebral artery (×40), **(B)** Alveolar septum(×200), **(C)** Myocardiim (×100), **(D)** Gastrointestinal tract (×40), **(E)** Subcapsular and peripapillary pancreas (×100), **(F)** Adrenal gland (×40), **(G)** Left renal cortex (×200), **(H)** Uterine wall (×100), **(I)** Lymph nodes (×200).

The trends of the patient’s critical laboratory results, significant clinical events, and main therapeutic interventions are illustrated in **Figure 2**. The patient was admitted with autoimmune hemolytic anemia (AIHA), treated initially with methylprednisolone (MP) 40mg once daily. From Day 2, intravenous immunoglobulin (IVIG) 20g was added once daily for 3 days. On Day 4, transthoracic echocardiography revealed right ventricular thrombus (**Figures 2B**, **C**), combined with positive antiphospholipid antibodies (aPL), raising suspicion of antiphospholipid syndrome (APS). This prompted intensification of immunosuppressive therapy (MP 40mg twice daily) and initiation of argatroban anticoagulation. On Day 6, clinical deterioration with markedly elevated SC5b-9 levels, suggesting catastrophic APS (CAPS). MP was escalated to 1g once daily (pulse therapy for 3 days), then reduced to maintenance dose (40mg once daily). From Day 7, adjunctive plasma exchange (PEX) was performed once daily for 3 days. Following sedative cessation, the patient remained comatose wit), left internal carotid artery thrombosis (**Figure 2E**), and (3) tonsillar herniah fixed pupillary dilation. On Day 11, the head and neck computed tomography angiography (CTA) demonstrated (1): acute subarachnoid hemorrhage (**Figure 2D**) (2tion. Anticoagulation was immediately discontinued.

Right heart overload necessitated norepinephrine (NE) infusion (0.4 μg/kg/min), with levosimendan added on admission for inotropic support. On Day 2, worsening right heart failure prompted NE dose escalation. After CTPA excluded PE, the patient received inhaled nitric oxide (NO) for pulmonary hypertension. Subsequent improvement in systemic perfusion permitted transition to oral tadalafil and ambrisentan on Day 6.

Empirical antimicrobial therapy with meropenem was administered. Following high-dose glucocorticoid pulse therapy, the patient developed persistent fever and neutropenia on Day 10, necessitating the addition of teicoplanin to broaden Gram-positive bacterial coverage. On Day 11, subsequent cultures isolated multidrug-resistant (MDR) *Klebsiella pneumoniae* (*K. pneumoniae*) and *Acinetobacter baumannii* (*A. baumannii*) from bronchoalveolar lavage fluid (BALF), with *Enterococcus faecium* bacteremia. The targeted therapy was performed with ceftazidime-avibactam, polymyxin B, and continued teicoplanin.

Following Day 12, the patient exhibited progressive respiratory failure necessitating advanced ventilatory support with profound circulatory instability. Despite maximal interventions, the patient succumbed on Day 22 of admission.

**Figure 4:**
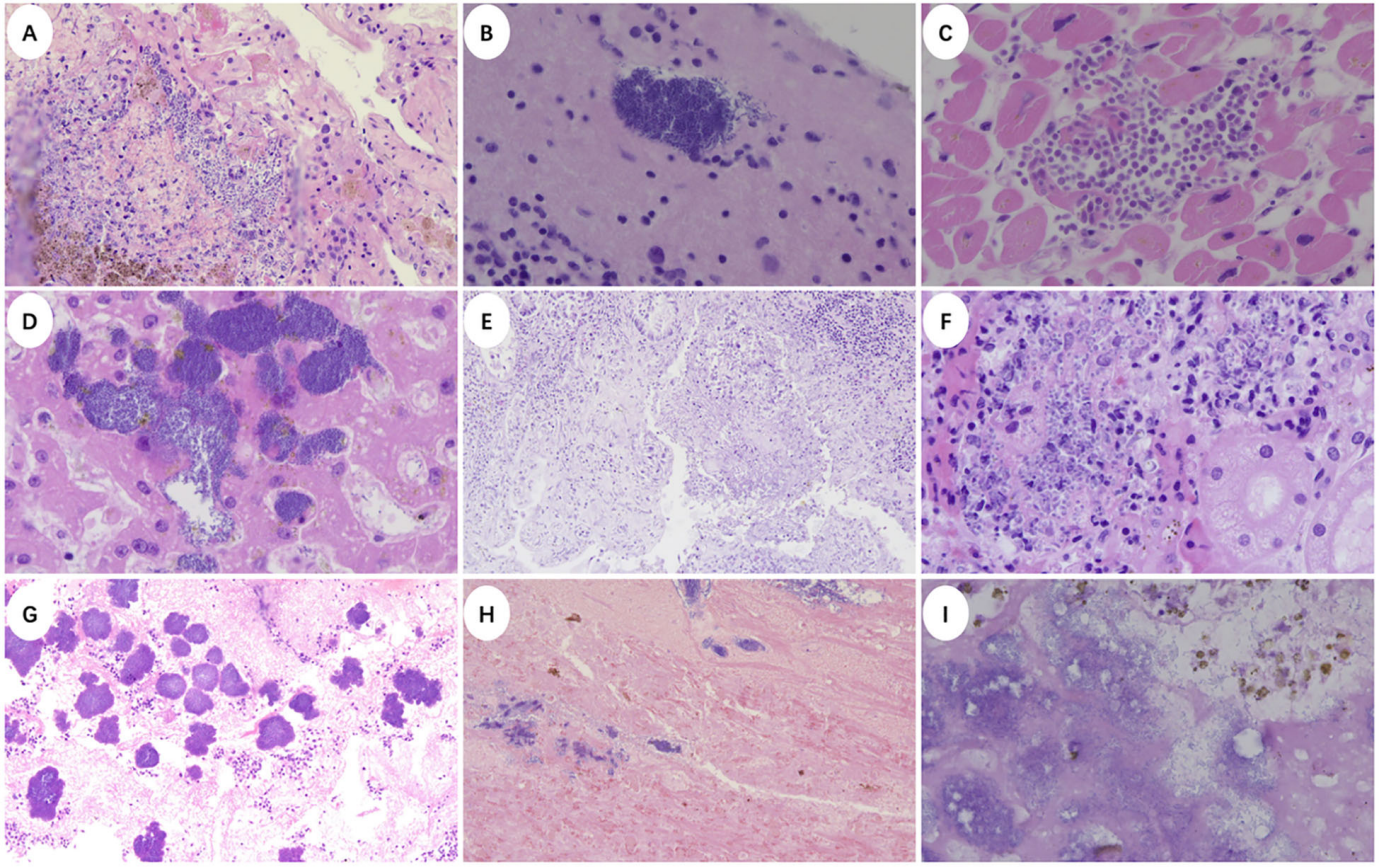
Clinicopathological findings of disseminated infection. **(A)** *Aspergillus* flora in the lungs (×200). **(B)** Heart valve vegetation (×400). **(C)** Myocardial fungal spores (×400). **(D)** Bacterial flora in liver parenchyma. (×400) **(E)** *Aspergillus* flora in the mucosa of the gastric cardia (×100). **(F)** Fungi flora in the cortex of the left kidney (×400). **(G)** Endometrial bacterial flora (×100). **(H)** Cerebellar fungal flora (×100). **(I)** Brain fungal flora (×400).

We conducted a histopathological analysis of the patient’s postmortem tissue samples after obtaining written informed consent from the patient’s family. Thrombosis were found in brain, heart, lung, gastrointestinal tract, pancreas, adrenal gland, renal, uterine and lymph nodes (**Figure 3**). The presence of widespread thrombosis in multiple organs supported the diagnosis of CAPS. In addition, bacterial or Fungi flora were identified in lungs, myocardial, liver, kidney, endometrial, cerebellar and brain (**Figure 4**). Subsequently we performed metagenomic next-generation sequencing (mNGS) on critical organs including the heart, lungs, and brain (**Table 2**). The analysis identified *Aspergillus* sequences in all three tissue types, multiple bacterial pathogens in both intracranial and cardiac specimens, including *Acinetobacter baumannii*, *Escherichia coli*, *Enterococcus* spp., *Klebsiella pneumoniae*, and *Staphylococcus aureus*, collectively confirming the diagnosis of severe disseminated infection.

**Table 2:** Tissue mNGS-DNA read counts.

Tissues	*Aspergillus flavus*	*Aspergillus fumigatus*	*Aspergillus niger*	*Acinetobacter baumannii*	*Escherichia coli*	*Enterococcus* *facecalis*	*Enterococcus* *facecium*	*Klebsiella pneumoniae*	*Staphylococcus aureus*
Lung	25	ND	73	ND	ND	ND	ND	ND	ND
Heart	ND	20	22	3080	ND	4562	3772	30924	ND
Brain	6	ND	ND	ND	9791	ND	6400	62778	4087

mNGS, Metagenomic Next-Generation Sequencing, ND, Not Detected.

Therefore, the patient was ultimately diagnosed with CAPS and died of disseminated infection.

## Discussion

In this case, the patient initially presented with autoimmune hemolytic anemia and thrombocytopenia. However, the cause of neurological involvement was unknown at onset. Thrombotic microangiopathy was suspected, including thrombotic thrombocytopenic purpura (TTP), atypical hemolytic uremic syndrome (aHUS), and disseminated intravascular coagulation (DIC), which were ruled out by normal ADAMTS13 activity and negative inhibitors, normal CFI, CFH, and CFB, and normal coagulation tests, respectively. Hemophagocytic Lymphohistiocytosis (HLH) was also carefully considered as a differential diagnosis given the presence of persistent fever, extreme hyperferritinemia and cytopenias. However, the absence of splenomegaly, hypofibrinogenemia, and bone marrow hemophagocytosis made HLH less likely (9). With the detection of positive LA and aCL-IgG, along with a tricuspid valve thrombus, a diagnosis of APS was considered. Given the disease course of less than 12 weeks, confirmatory testing for persistent antibody positivity was not feasible, and the aCL-IgG titer was low; thus, the antibody profile did not meet classic APS criteria. Drawing on experience from previously reported cases of seronegative APS (6–8), APS remained highly suspected, and with rapid disease progression, the development of CAPS was considered. This suspicion was confirmed by autopsy, which revealed extensive thrombosis in multiple organs, including the brain, heart, and kidneys.

A definite diagnosis of CAPS is made when all four diagnostic criteria are present: ① involvement of three or more organs/tissues; ② development of manifestations in less than a week; ③ histological evidence of intravascular thrombosis; and ④ presence of antiphospholipid antibodies on two occasions six weeks apart. A probable diagnosis of CAPS is established when a combination of these criteria is present (3, 4). CAPS affects a variety of organs and tissues, particularly kidneys, lungs, central nervous system, heart, skin, liver, and gastrointestinal tract (10, 11). Approximately 18% of patients present with renal disease as their initial symptom. The most common renal manifestations are hypertension, proteinuria, hematuria, and acute renal failure (12). Central nervous system is involved in 62% patients. Heart is involved in almost half of patients (13), and thrombotic heart valve lesions with sterile vegetations are occasionally presented. Lungs are involved in 24% patients, with rarely thrombosis of pulmonary arteries and arterioles.

In this patient, thrombosis was presented in tricuspid valve, carotid artery, and furthermore, microthrombosis was found in brain, heart, lung, gastrointestinal tract, pancreas, adrenal gland, renal, uterine and lymph nodes. Therefore, a diagnosis of CAPS was established in this patient. It is noteworthy that there were discrepancies between the imaging findings during his lifetime and the autopsy results. Early contrast-enhanced CT and ultrasound examinations of the portal vein, renal arteriovenous system, and peripheral vessels did not reveal any clear thrombosis. Tricuspid valve and carotid artery thrombosis were also only discovered in the later stages of disease progression. The most plausible explanation may be that CAPS-related thrombosis primarily exhibit microvascular characteristics, with volumes below the detection threshold of conventional imaging techniques.

In addition, the differential diagnosis for thrombosis included coronary artery thrombosis and septic thrombosis. Markedly elevated troponin, absence of ECG changes localized to specific coronary territories, and autopsy findings of widespread microvascular thrombosis (without macroscopic coronary thrombosis) support microvascular thrombosis rather than epicardial coronary thrombosis as the mechanism of myocardial injury. Septic thrombosis warranted careful consideration in the differential diagnosis. No thrombus specimen was available for analysis during early disease. Although tricuspid and carotid artery thrombi became visible on later imaging, antemortem sampling was not feasible due to the high-risk locations and the patient’s clinical instability. By the time of death, the patient had developed severe secondary infection, making postmortem analysis unreliable for determining whether thrombi were primarily infective. However, three negative blood cultures early in the course, absence of abscess on imaging, and autopsy findings of widespread microvascular thrombosis—consistent with CAPS rather than infective thromboembolism—suggest septic thrombosis is unlikely.

The recommendations for initial treatment of CAPS are triple-therapy with anticoagulation, corticosteroids, and PEX or IVIG (8, 14), which has the highest survival than each monotherapy (15).

After confirming tricuspid valve thrombosis, the anticoagulant therapy was immediately started. In this case, we used new anticoagulant drug argatroban, but not heparin or low molecular weight heparin, because severe thrombocytopenia and high risk of bleeding. We balanced the bleeding risk by controlling the APTT at 35–40 seconds. However, the patient developed subarachnoid hemorrhage (SAH), and anticoagulation therapy was forced to discontinue. SAH is a rarely reported neurological complication of CAPS (16, 17). The pathogenesis of such bleeding events is likely multifactorial, involving antiphospholipid antibody–mediated endothelial injury, dysregulated coagulation, thrombocytopenia-related hemostatic impairment, secondary inflammatory vascular damage, and anticoagulation therapy Chronic thrombotic microangiopathy may predispose APS patients for both ICH and SAH in combination with other CVD risk factors (included hypertension and platelet disorders) (18). In our patient, the coexistence of diffuse microvascular injury, dysregulated coagulation, carotid artery thrombosis, cerebral edema, and systemic anticoagulation likely acted synergistically to increase the risk of SAH. Microthrombi were observed in the adipose tissue surrounding spleen, pancreatic, adrenal glands, lymph nodes, and the left middle cerebral artery, suggesting that thrombus storm was not effectively prevented. Asherson and colleagues find that only anticoagulants significantly reduces death in a large study of CAPS and another study shows that anticoagulants is the top beneficial effect on prognosis (19). Furthermore, in successfully treated cases, patients ultimately received anticoagulant therapy, which may are temporarily suspended due to bleeding (2). These findings indicate that anticoagulant therapy is indispensable for treatment of CAPS. Further clinical trials are required to determine the efficacy of argatroban in CAPS comparing with unfractionated heparin or low-molecular-weight heparin.

The patients were aggressively treated with IVIG, PEX and methopredinisolone pulse therapy, to remove antiphospholipid antibodies and activated complements from plasma, with a modest improvement in thrombocytopenia. However, multidrug-resistant severe pneumonia and sepsis were complicated, which induced bone marrow suppression and rapid declined white blood cell and platelet counts. Despite extensive anti-infective treatment regimen, the patient ultimately died from severe infection and multiple organ failure. The autopsy revealed multi-organ bacterial and fungal infections. Therefore, infection is a major adverse prognosis factor for CAPS, in light of intensive immunosuppressive treatment. Prophylaxis of infections should be considered in CAPS patients, especially for patients with leukocytopenia.

Beyond standard therapy, anti-CD20 antibodies (e.g., rituximab) or complement-targeting agents (e.g., eculizumab) are reserved for refractory cases (20). Several reports suggest eculizumab is effective for CAPS patients unresponsive to standard therapy, especially those with complement-mediated thrombotic microangiopathy (21, 22). Marianna Strakhan and colleagues use eculizumab in early CAPS without heparin, and no serious side effect is observed. However, the rapid onset of severe infections eliminated the option of biologics in our patient. Early administration of these biologics might change the course of CAPS.

## Conclusion

We presented a rare case of CAPS patient featured with multi-organ thrombosis including brain, heart, lung, and gastrointestinal tract, pancreas, adrenal gland, renal, uterine and lymph nodes. Early diagnosis and aggressive triple treatment of anticoagulation, corticosteroids, and PEX or IVIG is essential for management of CAPS, and prophylaxis of infection might be a key factor to optimize the outcome of CAPS.

## Data Availability

The original contributions presented in the study are included in the article/supplementary material. Further inquiries can be directed to the corresponding authors.
